# Supplementary material for: A novel open-source cultivation system helps establish the first full cycle chemosynthetic symbiosis model system involving the giant ciliate Zoothamnium niveum
Source: Front Microbiol. 2024 Dec 12;15:1491485. doi: 10.3389/fmicb.2024.1491485 (PMC11669664; doi:10.3389/fmicb.2024.1491485)
Supplement: Supplementary file 1 [file Supplementary_file_1.pdf]

Supplementary Material :

Supplementary movie 1 - Timelapse photography capturing the first 10 hours of growth of a colony of *Z. niveum*. The timelapse starts right after the swarmer settled on a side of the chamber, differentiated into a terminal zooid and started to produce a stalk. During the first 1h40min, the stalk grows without symbionts, with the terminal zooids undergoing rotations. After that time, there is a pause in the stalk elongation. The terminal zooid changes its behaviour and after approximately 50 min it sheds a coat., the growth resumes, with the symbionts now covering the entire colony, including the stalk.

Supplementary movie 2 - Timelapse photography, 105 minutes long, of 5 *Zoothamnium niveum* colonies after 8 days of cultivation showing their contraction behaviour. Note that the cultivation setup is well suited for live imaging of the symbiosis and using a simple dissecting microscope and with a camera, quantitative data can easily be produced. Here for instance colonies from left to right contract at a frequency of 0.29, 0.26, 0.3, 0.28, 0.29 contractions/min respectively with a mean 0.284±0.0152.

This file has been converted from its original format for security purposes. Please use C70FFA7E6CEB5 as a reference.

Supplementary table 1 - Documented Sampling Sites and Environmental Contexts of *Zoothamnium niveum*. The table lists all known sampling locations for *Zoothamnium niveum*, detailing the geographic sites, coordinates, depths, and environmental substrates where specimens have been collected.

Number	Localisation	GPS point	Depth (meters)	Environment	Substrate	Reference
1	Twin Cays Island			Shallow water, tidal channel cut into mangrove peat	rocks	Rinke et al., 2006
2	Florida keys, Gulf of Mexico				rotting plant material	Bauer-Nebelsick et al., 1996a
3	Guadeloupe, French West Indies	16°N, 61.5°W	0.5-1.5 m	Shallow water, on mangrove sediment	sunken wood and leaf debris	Laurent et al., 2009, 2013
4	Canary Islands, Lanzarote	28°55'05''N - 13°39'36''W	6 m	On the sides of a rocky depression	rocks	Wirtz and Debelius, 2003
5	Corsica, France			Near *Sargassum* spp. debris accumulation	rocks	Bright et al., 2014
6	Adriatic sea				sunken woods	Bright et al., 2014
7	Red sea				rotting plant material	Bright et al., 2014
8	Tokyo bay			Deployed whale fall	bones	Kawato et al., 2010
9	White Point Beach California			Tidal pool close to coastal hydrothermal vents		Volland J-M., personal observation
10	Florida keys, Indian River Lagoon; East coast	27°25'11.78"N, 80°16'30.575"W			wooden debris	Indian River Lagoon Species Inventory; Clamp and Williams, 2006
11	Madeira Island			At the bottom of two large pools, Praia Piscina		Wirtz unpublished
12	São Tomé Island	0°01´25´´ N, 6°30´45´´ E	1.5 m		rotting coconuts	Wirtz, 2018
13	Gabon, coastal area	0°36´64´´N, 9°18´43´´E	1 m			Wirtz & Serval-Roquefort., 2020
14	Giglio Island, Western Mediterranean	42°22'01''N - 10°53'33''E	30 m	On sandy bottom	rocky tree trunk	Wirtz, 2008
15	Cyprus Island	34°57'45''N, 34°04'26''E		In a cave, close to cracks in the wall	rock wall	Wirtz, 2008

This file has been converted from its original format for security purposes. Please use C70FFA7E6CEB5 as a reference.

This file has been converted from its original format for security purposes. Please use C70FFA7E6CEB5 as a reference.

Supplementary Table 2 - comparison of the genome statistics of *Zoothamnium niveum*'s symbiont, *Ca*. Thiobius zoothamnicola, isolated from California and from the Caribeean (Belize and Guadeloupe). The three draft genomes have similar statistics. GC Content Contiguity and Quality of Assemblies: The number of contigs and the N50 values provide metrics for contiguity and quality of the genomic assemblies. All locations demonstrate high-quality assemblies with similar numbers of contigs (46 for Belize and Guadeloupe, and 45 for California) and substantial N50 values, indicating that the assemblies are well suitable for genomic investigations.

Completeness and Contamination: Completeness of the genomes is high across all samples, with Belize and Guadeloupe showing equal completeness estimates at 96.0%, while California shows slightly lower estimates at 85.5%. Heterogeneity: All samples exhibit zero percent estimated heterogeneity, suggesting that the assemblies represent single strains without significant intra-sample genetic diversity.

	Ca. Thiobius zoothamnicola		
	Belize	Guadeloupe	California
Assembly size (bp)	2,381,364	2,369,374	2,151,318
GC content (%)	49,40	49,60	49,52
nbr. of contigs	46	46	45
N50 (bp)	98,217	73,149	69,285
Completeness (%)	96.0	96.0	85.5
Contamination (%)	0.1	0.4	0.0
Heterogeneity (%)	0.0	0.0	0.0

This file has been converted from its original format for security purposes. Please use C70FFA7E6CEB5 as a reference.

Supplementary table 3 - Information about the seven genome bins obtained from the *Zoothamnium niveum* metagenome, including the Genome Taxonomy Database taxonomic assignment, the total size of the bin assembly, the number of contigs, the GC percentage, the estimated completeness, the estimated contamination level, the number of predicted genes, and the average protein length. The first bin is a taxonomic assignment to the genus *Aliishimia*. Bin 6, and 7 do not have a taxonomic assignment with a completeness below 0.1%. Bin number 3 contains eukaryotic sequences and corresponds to the ciliate host. Bins 3 and 4 are gammaproteobacterial draft genomes. Only one of these bins unambiguously emerged as the host's symbiont with an ANI of 96.7% compared to the published genome *Ca*. Thiobios zoothamnicola.

Bin #	Taxonomic Assignment	species	size (bp)	contig#	GC (%)	est. Comp. %	cont. %	predicted genes	Aver. Prot length (aa)
1	f__Rhodobacteraceae;g__Aliishimia		2,168,570	230	54.5	79.7	0.1	2,241	279
2	none		426,229	81	36.5	0.9	0.0	531	83
3	Eukaryote (Blast n with selected genes)	*Z. niveum* (host)	38,645,428	697	33.9	54.8	62.8	56,610	85
4	f__Thiotrichaceae		2,946,997	69	40.8	94.3	1.1	2,534	337
5	f__Sedimenticolaceae;g__VMDJ01	*T. zoothamnicola* (symbiont)	2,151,318	45	49.5	85.5	0.0	2,137	303
6	none		204,369	50	33.3	0.0	0.0	204	75
7	none		546,988	87	34.2	0.0	0.0	307	86

This file has been converted from its original format for security purposes. Please use C70FFA7E6CEB5 as a reference.

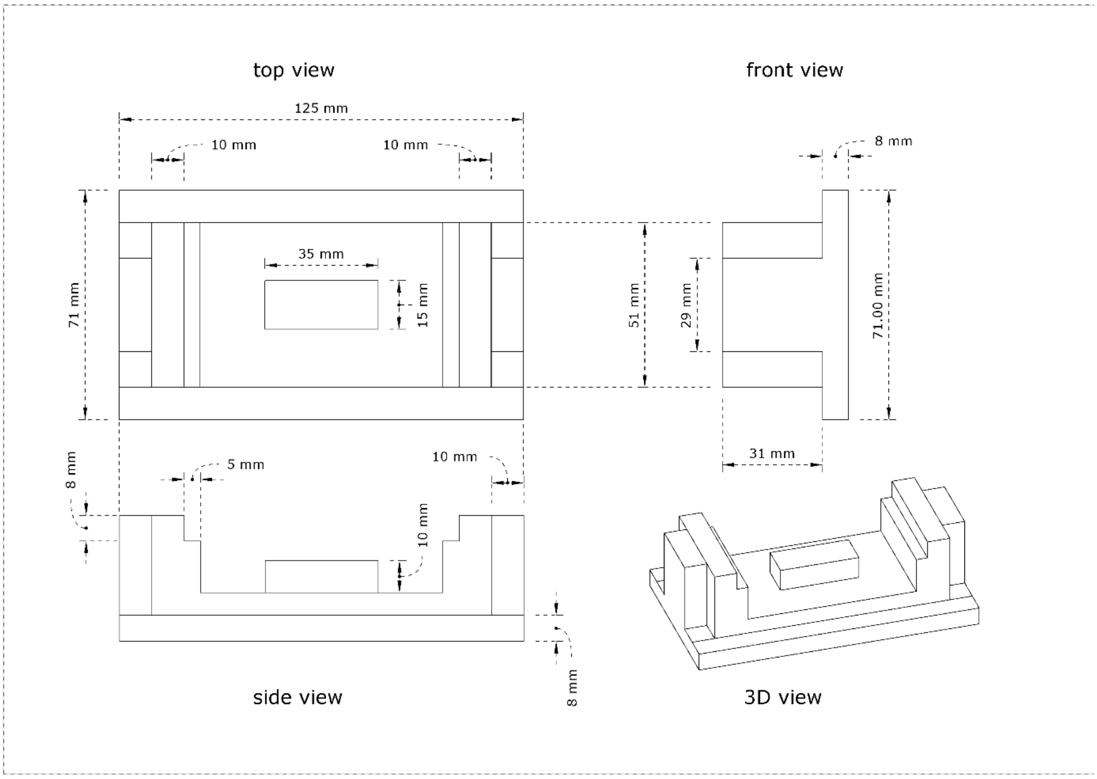

Figure S1 – Technical drawings of the central part of the mold. Top view, front view, side view, and 3D view of the central section of the mold used for casting the PDMS cultivation chamber. The stl file of this 3D model is available for download at https://github.com/jvolland/Zoothamnium-Cultivation-System.

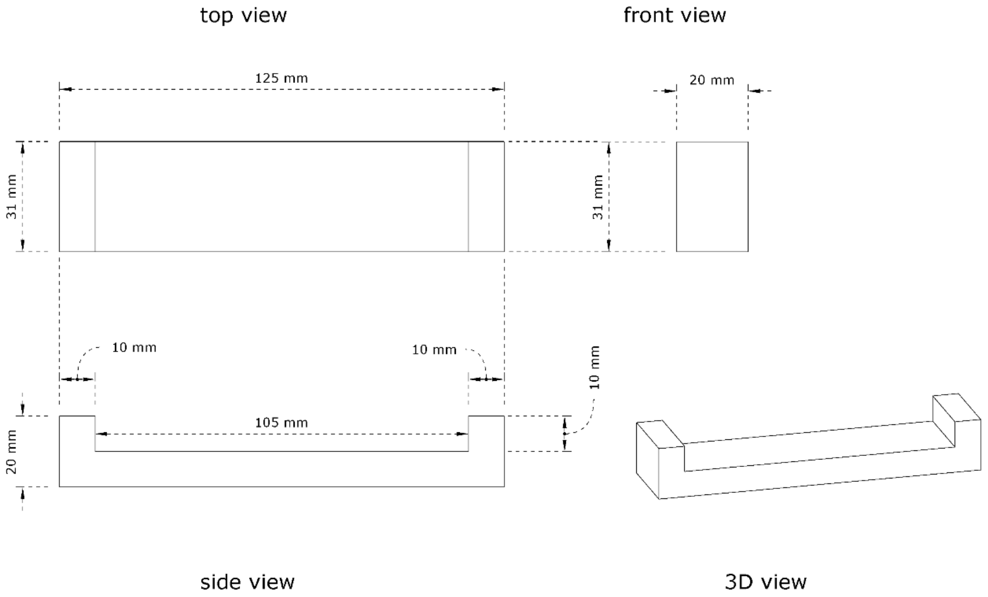

Figure S2 – Technical drawings of the side part of the mold. Top view, front view, side view, and 3D view of the side section of the mold used for casting the PDMS cultivation chamber. This side part is designed to close the two long edges of the central piece of the mold presented in Figure S1. The stl file of this 3D model is available for download at https://github.com/jvolland/Zoothamnium-Cultivation-System.

This file has been converted from its original format for security purposes. Please use C70FFA7E6CEB5 as a reference.

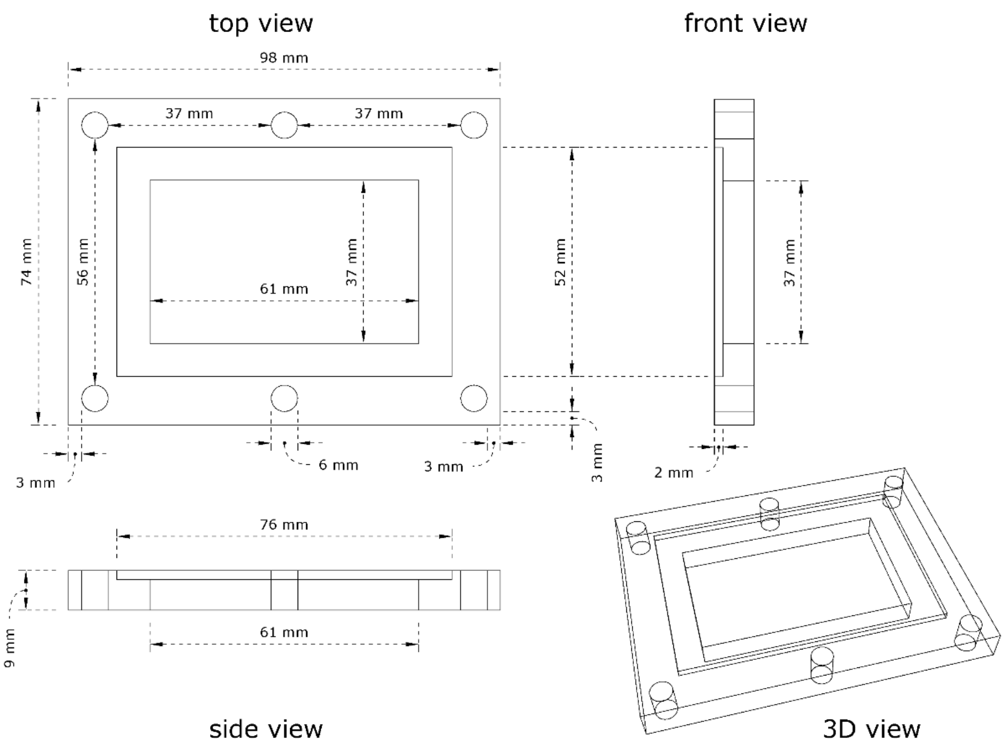

Figure S3 – Technical drawing of the cultivation chamber frame. Top view, front view, side view, and 3D view of the frame used for assembling the cultivation chamber. The frame secures the glass slide onto the PDMS body and maintains the alignment of the components. The top view shows the placement of six bolt holes that are used to hold the structure together, ensuring a tight and leak-free seal. The stl file of this 3D model is available for download at https://github.com/jvolland/Zoothamnium-Cultivation-System.

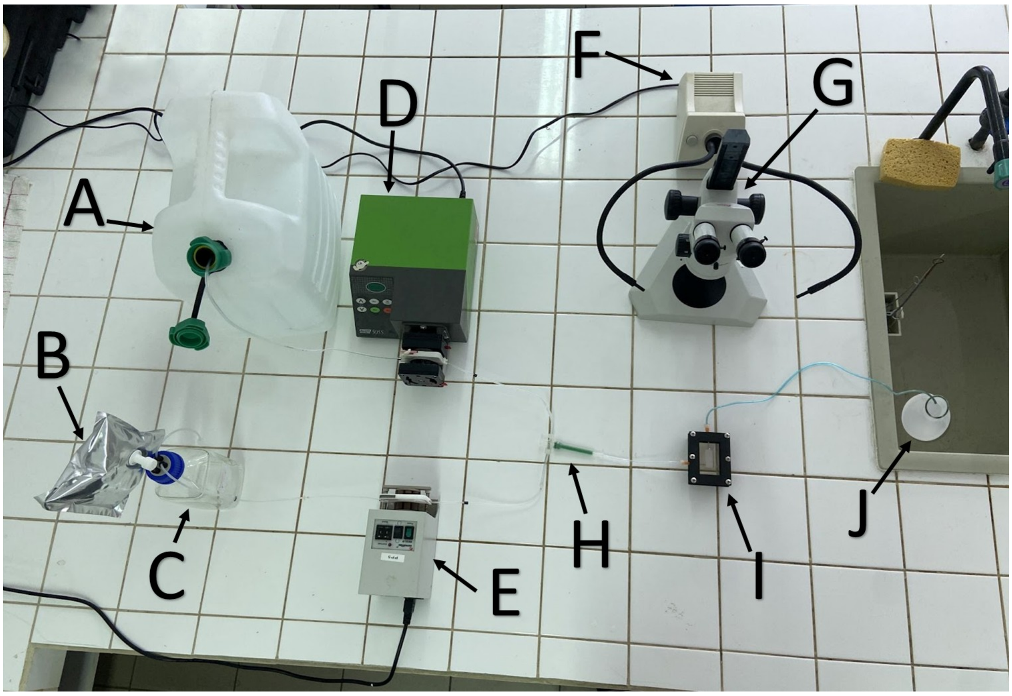

This file has been converted from its original format for security purposes. Please use C70FFA7E6CEB5 as a reference.

Figure S4 - Overview of the culture system. (A) filtered sea water tank; (B) gas bag filled with argon; (C) anoxic sulfide solution with 20gr/L NaCl; (D) peristaltic pump for sea water; (E) peristaltic pump for sulfidic water; (F) light source; (G) dissection microscope; (H) static mixer; (I) culture chamber; (J) outlet water collection.

This file has been converted from its original format for security purposes. Please use C70FFA7E6CEB5 as a reference.

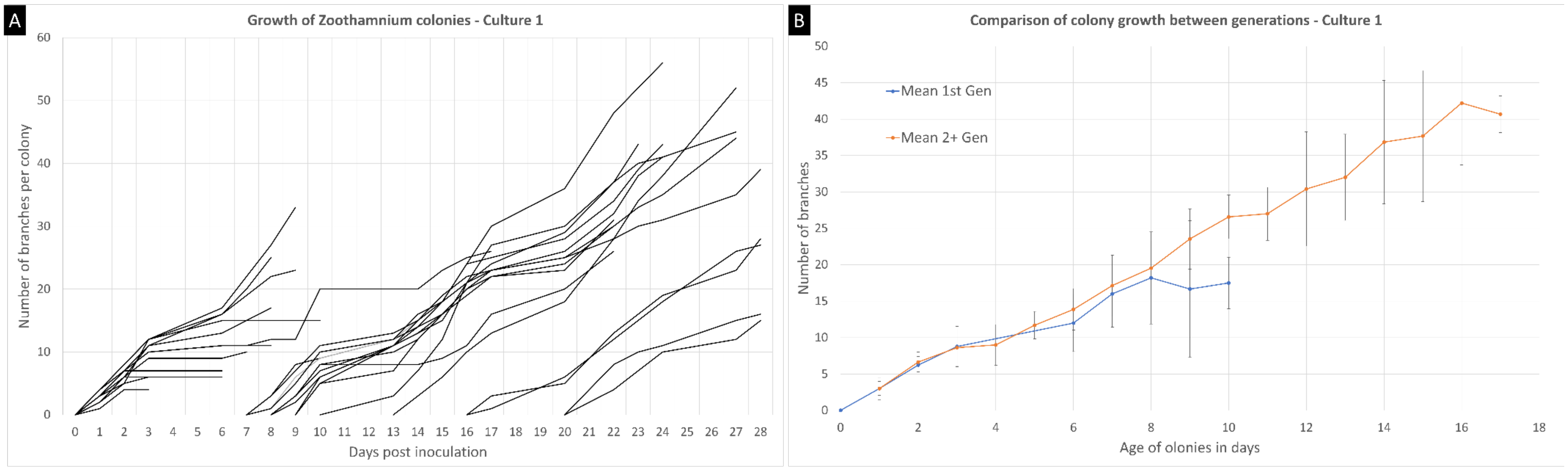

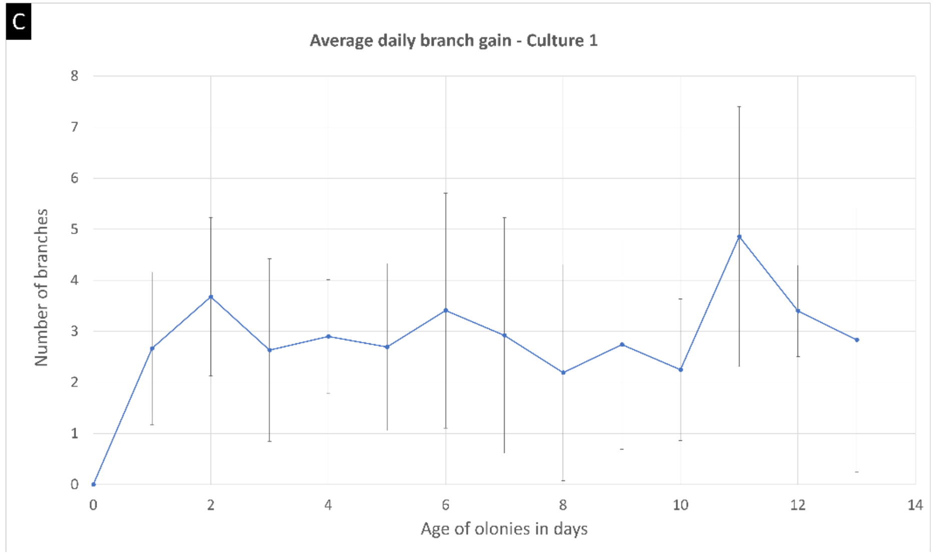

<u>Figure S5</u> - Growth of *Zoothamnium niveum* culture #1. (A) Growth of randomly selected individual colonies. The second generation of colonies start to grow after 7 days.; (B) Comparison of colony growth between the first generation (n=13) and the following generations (n=16); (C) Average branch gain per day measured on our culture (n=29).

This file has been converted from its original format for security purposes. Please use C70FFA7E6CEB5 as a reference.

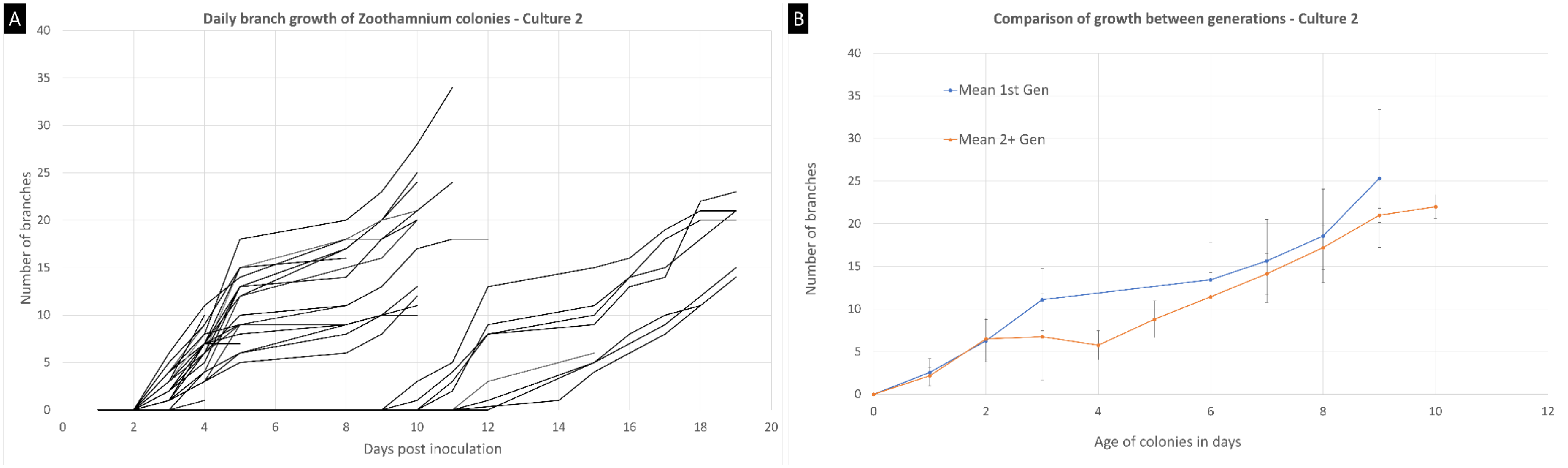

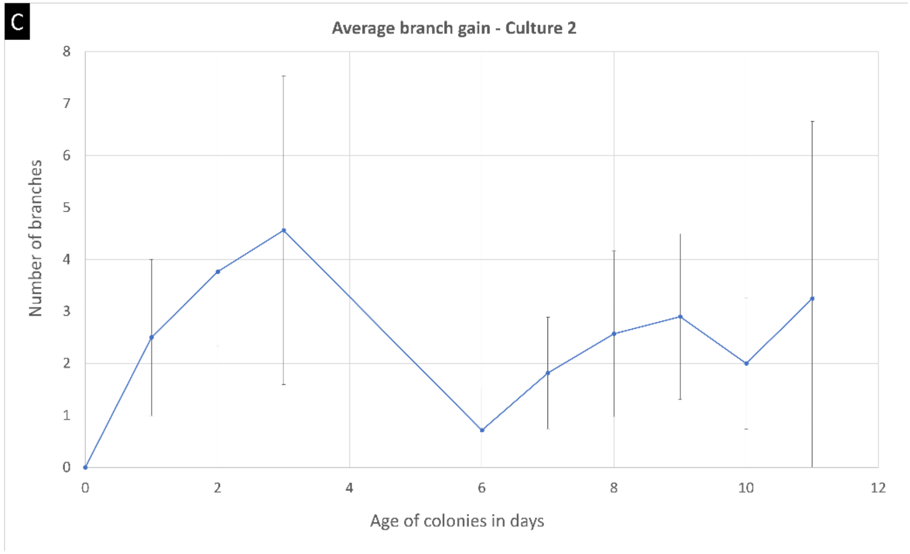

<u>Figure S6</u> - Growth of *Zoothamnium niveum* culture #2. (A) Growth of randomly selected individual colonies. The second generation of colonies start to grow after 9 days.; (B) Comparison of colony growth between the first generation (n=18) and the following generations (n=10); (C) Average branch gain per day measured on our culture (n=28).

This file has been converted from its original format for security purposes. Please use C70FFA7E6CEB5 as a reference.

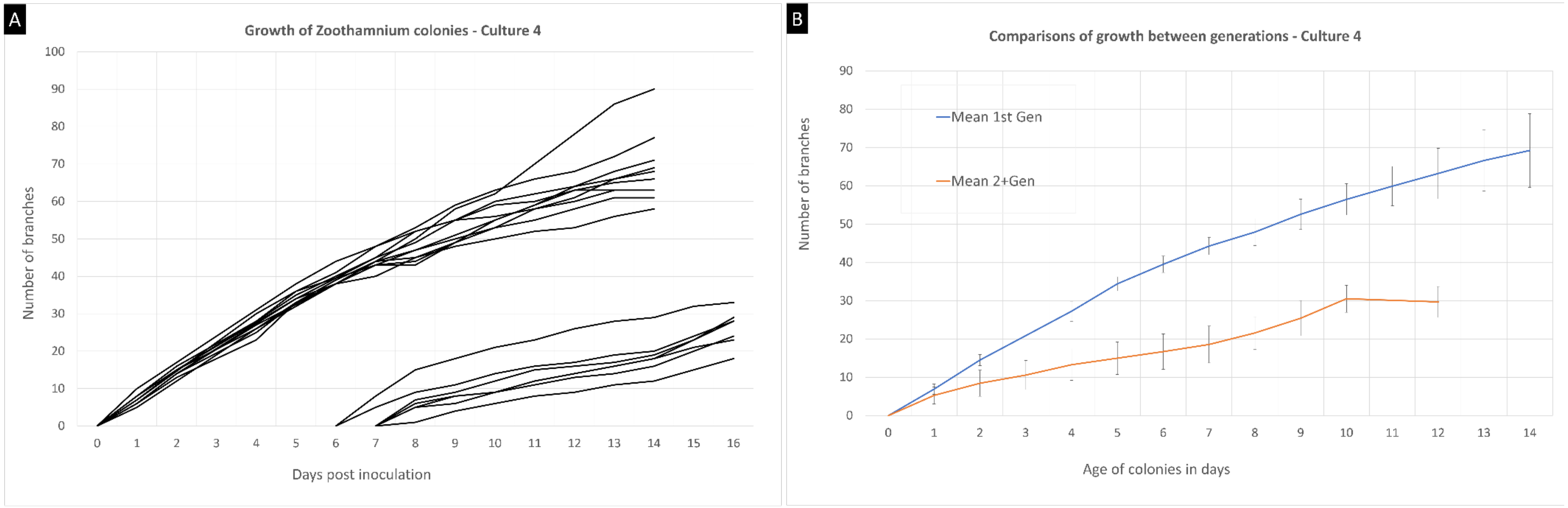

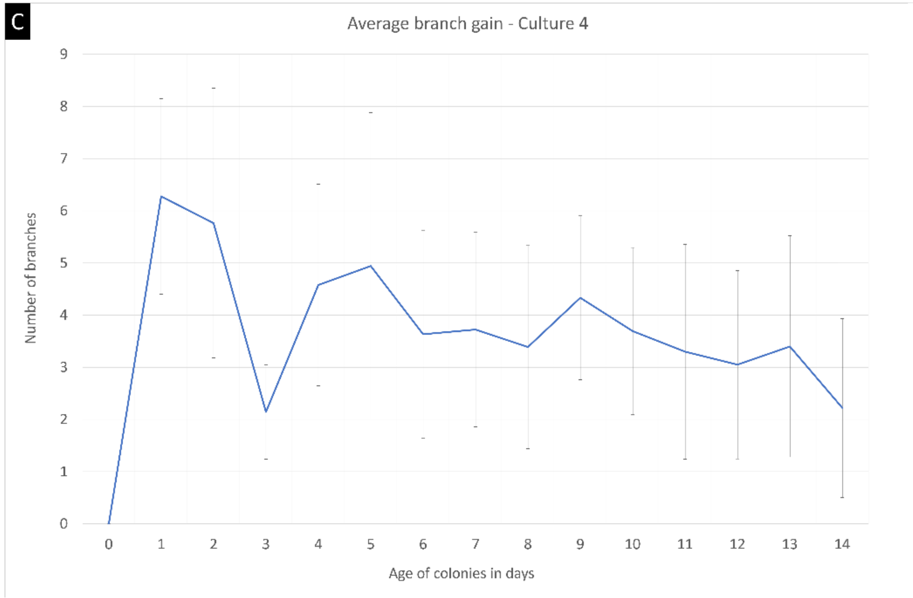

<u>Figure S7</u> - Growth of *Zoothamnium niveum* culture #1. (A) Growth of randomly selected individual colonies. The second generation of colonies start to grow after 7 days.; (B) Comparison of colony growth between the first generation (n=13) and the following generations (n=16); (C) Average branch gain per day measured on our culture (n=29).

This file has been converted from its original format for security purposes. Please use C70FFA7E6CEB5 as a reference.

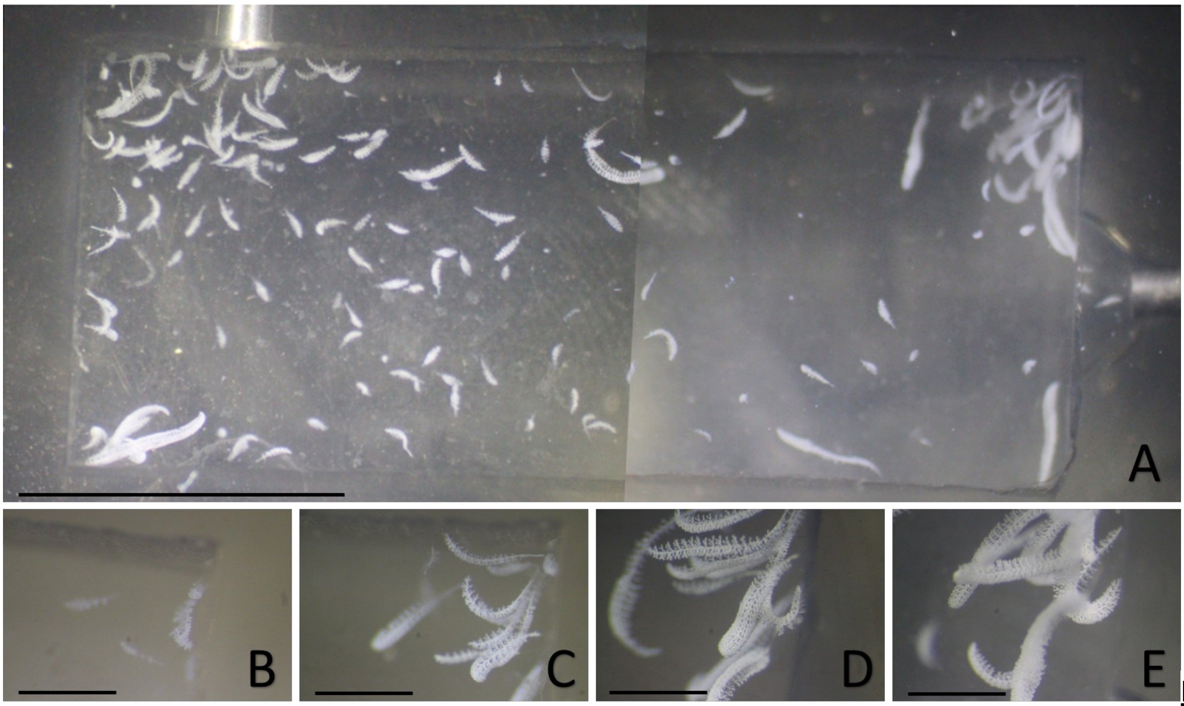

Figure S8 – Overview of culture #3. (A) Photo-montage of the whole culture chamber 15 days after incubation; scale bar = 1 cm. The detailed view of the top right corner is given at higher magnification and shows the growth of *Zoothamnium niveum* colonies after 1 day (B), after 5 days (C), after 10 days (D), and after 15 days of laboratory culture; scale bars = 0.25 cm.

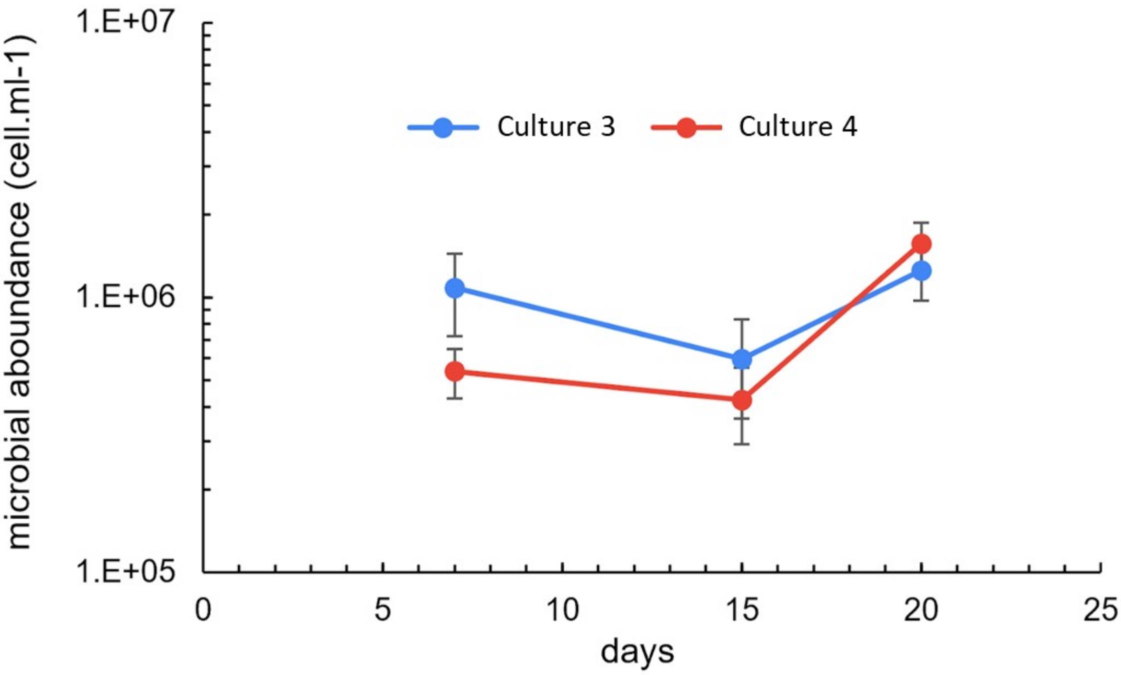

Figure S9 – Monitoring of microbial abundance in the outlet water of cultures 3 and 4.

This file has been converted from its original format for security purposes. Please use C70FFA7E6CEB5 as a reference.

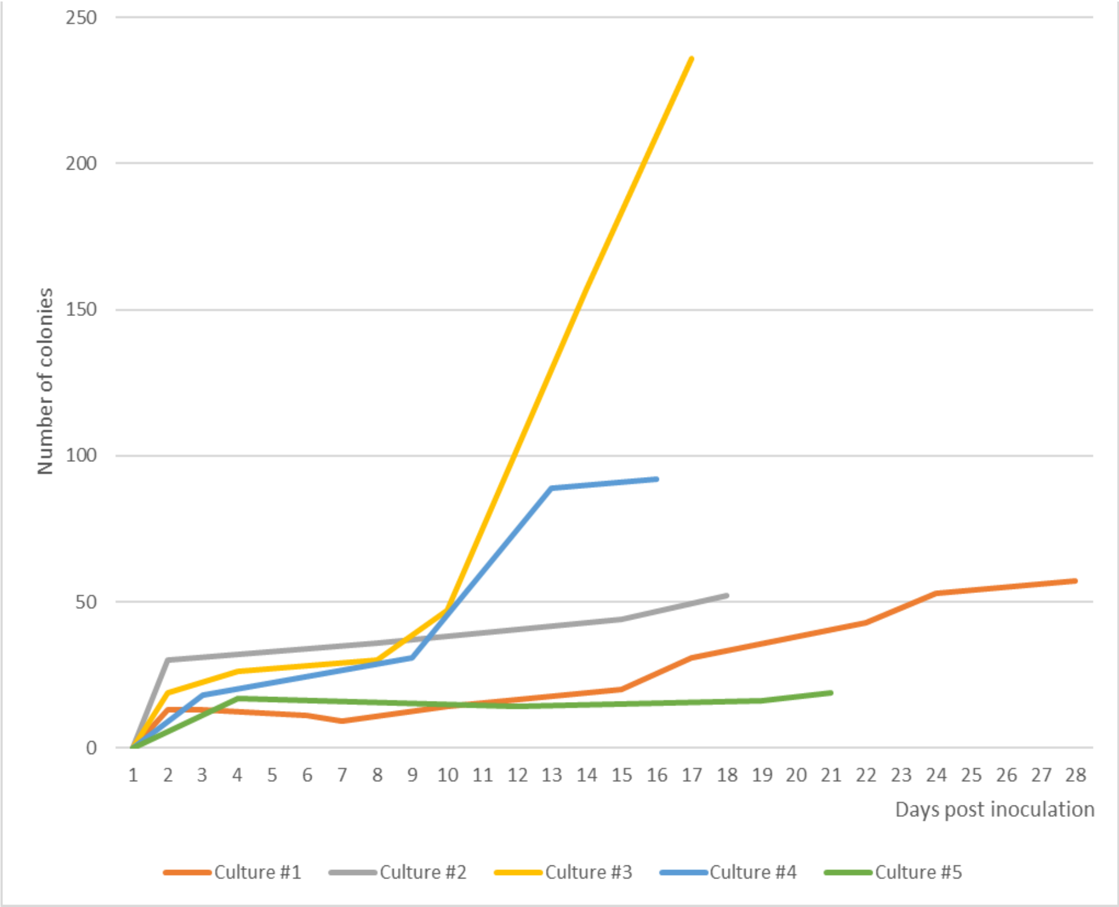

<u>Figure S10</u> – Evolution of the number of colonies within cultures. The number of swarmers injected in the chambers as inoculum and the percentage of settlements were as followed: culture #1: 38 swarmers with 34% settlement; culture #2: 68 swarmers with 44% settlement; culture #3: 82 swarmers 41% settlement; culture #4: 61 swarmers 51% settlement; culture 5: 19 swarmers.

This file has been converted from its original format for security purposes. Please use C70FFA7E6CEB5 as a reference.

Supplementary data 1 -*Ca*. Thiobius zoothamnicola's 16S rRNA gene sequence (sampled from White Point Beach, CA, USA).

>WPB_14_Thiobius_zoothamnicola16S
GAACTGAAGAGTTTGATCCTGGCTCAGATTGAACGCTGGCGGCATGCTTAACACATGCAAGTCGAA
CGCGAAAGCGCTTTCGGGCGTTAGTAGAGTGGCGGACGGGTGAGTAACGCGTGGGAATCTACCTG
ATAGTGGGGGATAACTCGGGGAAACTCGAGCTAATACCGCATACGCCCTACGGGGGAAAGACTTCG
GTCACTATCAGATGAGCTCGCGTCAGATTAGCTTGTTGGTGAGGTAATGGCTCACCAAGGCAACGAT
CTGTAGCTGGTCTGAGAGGACGAACAGCCACACTGGGACTGAGACACGGCCCAGACTCCTACGGGA
GGCAGCAGTGAGGAATATTGCACAATGGGGGAAACCCTGATGCAGCAATGCCGCGTGTGTGAAGA
AGGCCTGCGGGTTGTAAAGCACTTTCAGTTGGGAAGATAATGACGTTACCAACAGAAGAAGCACCG
GCTAACTCCGTGCCAGCAGCCGCGGTAATACGGAGGGTGCAAGCGTTAATCGGAATTACTGGGCGT
AAAGCGCGCGTAGGCGGCTAGGTCAGTCAGATGTGAAATCCCCAGGCTCAACCTGGGAATTGCATT
TGATACTGCTTGGCTAGAGTATGGTAGAGGGTGGTGGAATTCCAGGTGTAGCGGTGAAATGCGTAG
ATATCTGGAGGAACATCAGTGGCGAAGGCGGCCACCTGGATCAATACTGACGCTGAAGTGCGAAA
GCGTGGGGAGCAAACAGGATTAGATACCCTGGTAGTCCACGCCGTAAACGATGTCAACTAGCCGTT
GGGTTTTTATAAATTTAGTGGCGTAGCTAACGCGATAAGTTGACCGCCTGGGGAGTACGGCCGCAA
GGTTAAAACTCAAAGGAATTGACGGGGGCCCGCACAAGCGGTGGAGCATGTGGTTTAATTCGATGC
AACGCGAAGAACCTTACCAGTTCTTGACATCCAGTGAACTTTCCAGAGATGGATTGGTGCCTTCGGG
AACACTGTGACAGGTGCTGCATGGCTGTCGTCAGCTCGTGTCGTGAGATGTTGGGTTAAGTCCCGTA
ACGAGCGCAACCCTTGTCCTTAGTTGCCAGCACTTCGGGTGGGAACTCTAAGGAGACTGCCGGTGA
CAAACCGGAGGAAGGTGGGGATGACGTCAAGTCATCATGGCCCTTATGAACTGGGCTACACACGTG
CTACAATGGACGGTACAGAGGGCTGCAAACCCGCGAGGGGGAGCTAATCCCAAAAAACCGTTCGTA
GTCCGGATTGCAGTCTGCAACTCGACTGCATGAAGTCGGAATCGCTAGTAATCGCAGATCAGAATGC
TGCGGTGAATACGTTCCCGGGCCTTGTACACACCGCCCGTCACACCATGGGAGTGGGTTGCAAAAG
AAGTGGGTAGCTTAATAATGGGCGCTCACCACTTTGTGATTCATGACTGGGGTGAAGTCGTAACAA
GGTAGCCGTAGGGGAACCTGCGGCTGGATCACCTCCTTT

This file has been converted from its original format for security purposes. Please use C70FFA7E6CEB5 as a reference.

Supplementary data 2 - *Zoothamnium niveum*'s 18S rRNA gene sequence (sampled from White Point Beach, CA, USA).

>Zoothamnium_niveum_WPB_partial18S
AATCTGGTTGATCCTGCCAGTAGTCATATGCTTGTCTCAAAGATTAAGCCATGCATGTGTAAGTATAA
GTATTATACGGCGAAACTGCGAATGGCTCATTAAATCAGTTATAATTTATTTGATAATCGAAAGTTAC
ATGGATAACCGTGACAAATTACAGCTAATACATGCAGTCAGACCCGGTCCAAAGGTCGTAATTATTA
GTATTAAACCAATTCCTTCGGGAGTGTGATGAATCATAGTAATCGAACGAATCGCTAATCTTGCGAT
AAATCATTCAAGTTTCTGCCCTATCAGCTTTGGATGGTAGTGTATTGGACTACCATGGCAGTCACGG
GTAACGGAGAATTAGGGTTCGATTCCGGAGAGGGAGCCTGAGAAACGGCTACCACATCTACGGAA
GGCAGCAGGCGCGAAAATTGCCCAATCCTGACACAGGGAGGCAGTGACAAGAAATAACAACTCTC
GGTTTCCGAGAAGTGTAATGAGGATAATTTACAAACCTTACCGAAAGCAATTGGAGGGCAAGTCTG
GTGCCAGCAGCCGCGGTAATTCCAGCTCCAATAGCGTATATTAAAGTTGTTGCAGTTAAAAAGCTCG
TAGTTGAAATTCTGATTGTATTGGGCCTCAGCACTCGATGCGTAGGTACCAGCAGTCATCCGCTTGC
AAACATATGTTCTTCCTTAAACAGAAGACTATGTGAGTAAGCATTTTACCTTGAGAAAAACAGAGTG
TTCCAGGCAGGTTTGTCCGGAATGCATTAGCATGGAATAATAGAATATGACTGGAGTCTATTTATTG
GTTTGAGACTACAGTAATGATTAATAGGAACAATCGGGGGCATTGGTACTTGTCAGTCAGAGGTGA
AATTCTAGGATTTGACAAAGACTAACAAATGCGAAAGCATTTGCCAAGGATGTTTTCATTAATCAAG
AACGAAAGTTAGGGGATCAAAGACGATCAGATACCGTCCTAGTCTTAACTATAAACCATACCGACTC
GGATTCAGATGAATAATAAAGTTCATTTGGGACCGTAGGAGAAATCAAAGTCTTTGGGTTCTGGGG
GGAGTATGGTCGCAAGGCTGAAACTTAAAGGAATTGACGGTTTTGCACCACCATGGAGTGGAGCCT
GCGGCTTAATTTGACTCAACACTGGGAAACTCATCAGGGCAAGAAGATTTTAGGATTGACAGATTGA
GAGTTCTTTCTTGATTGGTCTAGTGGTGGTGCATGGCCGTTCTTAGTTGGTGGAGTGATTTGTCTGGT
TAATTCCGTTAACGAACGAGACCTTAACCTGCTAACTAGTATTGCTATCCACAATGGCAATTACTTCT
TAGAGGGACTATGTGATGCAATCACATGGAAGTTTGAGGCAATAACAGGTCTGTGATGCCCTTAGA
TGTCCTGAGCTGCACGCGCGCTACAATGACACATTCAACGAGCATTTCCTGATCCGAAAGGATTTGG
GTAATCTTTTCAGTATGTGTCGTGCTAGGGATAGATCTTTGTAATTGTGGATCTTGAACGAGGAATTC
CTAGTAAGCACAAGTCATCAGCTTGTGCTGATTACGTCCCTGCAAAATGTACACACCGCCCGTCGCT
GTTACCGATTGAGTGCTCAGGTGAACCTTCTTGATAGTGTCAAAACTAAAAATTAAGTAAACCTTAG
CACTTAGAGGAAACAAAAGTCGTAACAAGGTTTCCGTAGGTGAACCTGCGGAAGGATCATTT

This file has been converted from its original format for security purposes. Please use C70FFA7E6CEB5 as a reference.
